# Library Preparation and Multiplex Capture for Massive Parallel Sequencing Applications Made Efficient and Easy

**DOI:** 10.1371/journal.pone.0048616

**Published:** 2012-11-05

**Authors:** Mårten Neiman, Simon Sundling, Henrik Grönberg, Per Hall, Kamila Czene, Johan Lindberg, Daniel Klevebring

**Affiliations:** 1 Department of Medical Epidemiology and Biostatistics, Science for Life Laboratory, Karolinska Institutet, Stockholm, Sweden; 2 Department of Medical Epidemiology, Karolinska Institutet, Stockholm, Sweden; The Roslin Institute, University of Edinburgh, United Kingdom

## Abstract

During the recent years, rapid development of sequencing technologies and a competitive market has enabled researchers to perform massive sequencing projects at a reasonable cost. As the price for the actual sequencing reactions drops, enabling more samples to be sequenced, the relative price for preparing libraries gets larger and the practical laboratory work becomes complex and tedious. We present a cost-effective strategy for simplified library preparation compatible with both whole genome- and targeted sequencing experiments. An optimized enzyme composition and reaction buffer reduces the number of required clean-up steps and allows for usage of bulk enzymes which makes the whole process cheap, efficient and simple. We also present a two-tagging strategy, which allows for multiplex sequencing of targeted regions. To prove our concept, we have prepared libraries for low-pass sequencing from 100 ng DNA, performed 2-, 4- and 8-plex exome capture and a 96-plex capture of a 500 kb region. In all samples we see a high concordance (>99.4%) of SNP calls when comparing to commercially available SNP-chip platforms.

## Introduction

Since the introduction of massively parallel DNA sequencing, there has been a rapid adoption of the different technologies in the sequencing field. Resequencing of full human genomes and targeted sequencing of exomes have enabled discoveries of genes and altered pathways in both mono- and polygenic inherited diseases [1], [2], [3], [4], [5]. Even though amplification-free library preparation protocols are available [6], [7], the vast majority of sample preparation strategies for massively parallel sequencing rely on amplification by PCR. In order to prepare a sample for sequencing, genomic DNA is sheared and end-repaired after which common adapter sequences, often containing barcodes, are ligated onto each fragment. This step is critical as a low efficiency in the ligation step yields a low number of amplifiable DNA templates for the downstream PCR step. Inefficient ligation thus leads to a low number of unique molecules available for sequencing (i.e. a library with low complexity) relative to the amount of starting material. Obviously, the performance of the library preparation process determines the amount of input DNA required in order to produce a sufficiently complex end product for sequencing. In order to improve the yield, one needs to increase the efficacy within each step and/or reduce the total number of clean-up steps during the library preparation. Several slight increases in the yield of each enzymatic step have the potential to positively affect overall yield significantly. Clean-up steps are common sources of loss of material and reduction of overall library yield. A typical yield in a spin-column purification is 60–80% [8], [9], thus for library preparation protocols with three purification steps prior to PCR, these steps alone decreases the yield by 50–80%. Automated protocols circumventing spin columns have been devised [10], capable of handling large numbers of samples. An issue with these protocols is that robotics are necessary to reach a large throughput.

The traditional Illumina TruSeq library preparation requires 1 µg DNA [11] and several approaches have been devised to lower the necessary input amount. Currently, the use of *in vitro* transposition is the most effective way of building sequencing libraries, where whole-genome sequencing of human samples can be achieved with 50 ng of DNA. Furthermore, conventional T7-based linear amplification, commonly used for microarrays, has been adopted to obtain a more even amplification of ligated products [12]. However, it requires several clean-up steps prior to amplification, which reduce the complexity of the library. Due to the inherent nature of ligation of full-length complementary adapters, only 25% of ligated molecules will be available for linear amplification. In addition to this, the Klenow DNA polymerase exo^(−)^ enzyme, which is used for adenylation after end-repair, does not distinguish between different nucleotides. Therefore, only 1/16 of the starting molecules will carry the correct 3′ overhang (A in both ends) for ligation, if nucleotides from the end-repair are not removed prior to adenylation. Zheng and colleagues refined the library preparation for the 454 sequencer [13] and reduced the number in cleanup steps, using a Y-shaped adapter with complementarity only in the ligating end. In this approach each double-stranded DNA molecule can give rise to two template molecules in the PCR step [14].

Genome wide association studies (GWAS) has led to the identification of hundreds of gene loci associated with different phenotypic traits [15]. Recent pioneering work demonstrated the feasibility of targeted resequencing to identify causal variants in regions identified through GWAS [5]. As the cost of sequencing decreases the relative cost of performing targeted enrichment increases. Multiplexed capture, where samples are barcoded and then mixed and used in a single capture reaction reduces the relative cost of enrichment. It is also an attractive means for increased throughput, especially in laboratories without access to infrastructure allowing automation. When sequencing a large number of samples the use of DNA barcodes is the most common method to determine the origin of the reads [16], [17]. To circumvent the need of equal amounts of unique barcodes as samples in a mixture, the combination of two different barcodes can be used to decipher the origin of the reads [18], [19]. Rohland and Reich have developed a dual barcode based method for cost-effective and automatable library preparation for multiplexed capture [20] but it is dependent of relatively large amounts of starting material [21]. The use of two different barcodes at each end of a molecule is appealing, but has the drawback that misidentified molecules cannot be identified as any two combinations of the barcodes are valid combinations.

**Figure 1:**
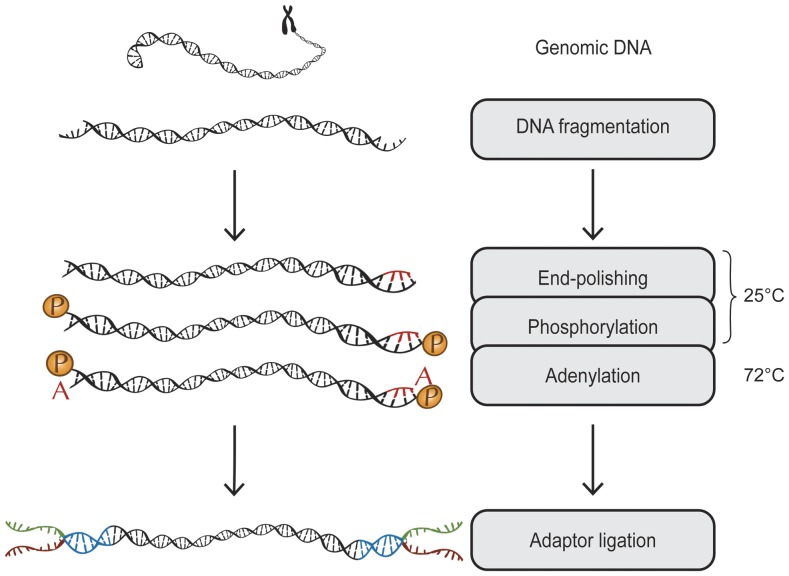
A schematic overview: genomic DNA is fragmentized, end-repaired, phosphorylated and adenylated in the same reaction. Adaptor ligation is followed by size-selection and PCR.

In order to perform parallel library preparation, we have devised a methodology, which only requires a single cleanup from fragmentation to PCR and where the entire enzymatic chain is functional in one single buffer (figure 1). By adjusting enzyme concentrations and changing the enzyme used in the adenylation step, a single combined size-selection and clean-up step using superparamagnetic beads is used in the procedure. This allows for cheap and easy automatable multiplex capture and sequencing, starting from small amounts of DNA.

## Materials and Methods

### DNA extraction

DNA was extracted from whole blood using Qiagen's QIAmp spin miniprep kit according to the manufacturers recommendations. The DNA concentration was measured using a Qubit fluorometer (Invitrogen, CA, USA) and the dsDNA HS kit.

### DNA fragmentation

Human genomic DNA was suspended in 120 µl nuclease free water and sheared using the Covaris (Covaris Inc, MA, USA) sonication system according to the manufacturers instructions. 1 µl of the sample were analyzed using an Agilent 2100 Bionalyzer (Agilent Technologies, Santa Clara, CA, USA) and the DNA 7500 kit.

### End-polishing, phosphorylation, adenylation and adaptor ligation

The fragmented DNA was transferred to a fresh 1.5-ml tube after which the volume was reduced to 30 µl using vacuum centrifugation. These 30 µl were mixed with 10 µl end-polishing/phosphorylation/adenylation mix to a final concentration of 1×T4 DNA ligase buffer, 4×0.5 mM dNTP, 0.25 mM ATP, 2.5% PEG 4000, 0.0025 U/ µl T4 DNA polymerase, 0.125 U/ µl T4 Polynucleotide kinase and 0.0025 U/ µl Taq DNA polymerase (recombinant) (all enzymes and buffers from Fermentas life sciences, Burlington, Canada). The DNA-samples were end-polished, adenylated and phosphorylated by incubating the reaction mixes for 15 min at 12°C, 15 min at 37°C, 20 min at 72°C and final 4°C forever in a pre-cooled thermal cycler (GeneAmp 9700 PCR system, Applied Biosystems). Ten microlitres of a ligation mix was added to the samples to a final concentration of 0.3 U/ µl T4 DNA Ligase and a 1∶10 molar ratio of DNA fragments to adaptor constructs (table S1). Adaptors were ligated to the template DNA by incubating the reaction mix at 16°C over night (16 h) in a pre-cooled thermal cycler (GeneAmp 9700 PCR system, Applied Biosystems).

### Short fragments removal

Short DNA fragments and unligated adaptor constructs were washed away by polyethylene glycol (PEG) mediated precipitation on carboxylic acid coated magnetic beads (MyOne, Invitrogen) using 6.3% PEG solution in a Magnatrix™1200 (NorDiag ASA, Oslo, Norway) liquid handling robot [22]. The μg-samples were split in 5 reactions prior to clean-up and the volumes were adjusted to 50 µl using 0.1×EB (Qiagen Elution Buffer). The DNA was eluted in 23 µl EB.

### Enrichment of ligated fragments

Barcoding and enrichment of ligated fragments was carried out by PCR. The eluted DNA was mixed together with PCR reagents and primers for a final concentration of 1xPhusion HF master mix (Finnzymes, Espoo, Finland) and 0.2 µM of each PCR primer (table S1). The reaction volume was 5×50 µl for the μg-samples and 50 µl for the ng-samples. The reactions were incubated in a thermal cycler (GeneAmp 9700 PCR system, Applied Biosystems) for 2 min at 98°C, 12 cycles of 10 s at 98°C, 30 s at 65°C, 20 s at 72°C and a final extension of 5 min at 72°C ending with an infinite hold at 4°C. Final library cleanup was done by PEG-mediated precipitation on carboxylic acid coated magnetic beads as described above. The final libraries were evaluated using an Agilent 2100 Bionalyzer (Agilent Technologies) and the DNA 7500 kit or the DNA High Sensitivity kit.

### Quanitative PCR

Quantitative PCR was carried out using the BioRad CFX96 instrument as instructed by the manufacturer. The function ratiocalc from the R-package qpcR [23], [24] was used to estimate the relative amounts of library molecules obtained from the different amounts of starting material. The function Cy0 was used to calculate Cy0-values, which correspond to the more traditional Ct-value but are more accurate [25].

### Enrichment of genomic regions

Samples prepared as described above from 100 ng or 1 µg DNA, were pooled for 2-, 4- and 8-plex exome capture. Exome capture was carried out using the SeqCap EZ Exome Library Version 1(Nimblegen) according to the manufacturers instructions with modified blocker oligonucleotides covering the entire Y-adapter. Equal amounts of each index-blocker were used, with a total of 1000 pmol per reaction (i.e. for the 2-plex 50 pmol of each of the two indices were used, for the 8-plex 125 pmol of each index was used). Post-capture PCR was run for 18 cycles.

### Adjustment for 96-plex library preparation and targeted resequencing

For the 96-plex capture reaction, 500 ng of DNA was mixed with 1.5 µl Fragmentase (NEB), 1.5 µ10× Fragmentase buffer and nuclease-free water to 15 µl. The reaction was incubated in 37°C for 20 minutes, followed by heat inactivation in 65°C for 15 minutes. Fragmented DNA was end-repaired, phosphorylated and adenylated by adding 5 µl master mix as described above. A double-stranded 8-bp barcode with an 3′ A overhang in one end and a 3′ 3-bp overhang in the other end was ligated the fragments in each well in the plate as described above (5′ ends were phosphorylated). Equal volumes of ligation mixture DNA from each well was pooled and cleaned up using PEG-mediated precipitation (see above). A modified Y-shaped adapter with a 3-bp overhang matching the one on the barcodes was ligated onto the pooled DNA after which unligated adapters were removed by PEG-mediated precipitation (see above). Pre-capture PCR was carried out as described above after which enrichment of a genomic region encompassing 500 kb was performed using a custom SureSelect XT kit (Agilent) according to the manufacturers instructions with the modification that the bait library was diluted a factor 10 prior to use. Post-capture PCR was performed as described above.

### Sequencing

Sequencing was carried out on the Illumina HiSeq 2000 system according to the manufacturers recommendations. All lanes were spiked with 1% phiX as a quality control.

### Low-level processing of sequence data and SNP calling

Raw data was aligned to the GRCh37 (hg19) genome using BWA (Burrows-Wheeler Aligner, version 0.5.9) [26]. Standard arguments were used except for –q 10, which soft-clips low-quality bases at the ends of reads. Tools available in the software suite Picard (http://picard.sourceforge.net) were used for quality control and removal of technical duplicates. Subsequently, the sequence data was realigned and base qualities recalibrated using the genome analysis toolkit (GATK) [27]. Single nucleotide polymorphisms (SNPs) were called with the MAQ SNP calling model, available in Samtools (version 0.1.16) [28]. To validate the SNP calls, the same DNA used for library preparation was assayed using the Affymetrix 6.0 SNP array. The Affymetrix data was processed as described previously [29]. For the 96-plex capture, the validation was carried out on the Illumina HumanHap300, 240 and 550 platforms as described previously [30].

### Ethics statement

This project was carried out according to the declaration of Helsinki. The Regional Ethics Committee in Stockholm specifically approved this study. Written consent was received from all participants of the study

## Results

To enable single-buffer library preparation, we replaced Klenow fragment exo(−) with Taq DNA polymerase as the adenylating enzyme. Taq has the propensity of remaining bound to the DNA if used in too high concentrations. As a consequence due to steric hindrance, the ligation will suffer from reduced efficiency. Therefore, we reduced the Taq DNA polymerase concentration in the adenylation step by a factor of 50 compared to recommended amounts, which improved the overall yield significantly (figure S1). To further increase the efficiency, we investigated the effect of prolonging the ligation time to two hours and over-night (16 h). We also investigated the effect of modifying the incubation temperature scheme during the end-polishing reaction for each enzyme by changing the traditional 30 min at 30°C into 15 min at 12°C (optimal for T4 DNA polymerase) plus 15 min at 37°C (optimal for T4 PNK). To investigate the importance of the three variables we prepared libraries from 100 ng DNA using all combinations of the variables and performed quantitative PCR (qPCR) on the ligation products (figure S2). An analysis of variance (ANOVA) table was constructed using the Cy0-values from the qPCR as outcome (table S2). The table shows that both the over-night ligation and the lowered DNA polymerase concentration have significant effects on the threshold cycle of the amplification, whereas the modified end-polishing incubation scheme shows no improvement in yield. We also investigated the fraction of duplicate molecules after sequencing for selected libraries, which shows a 10-fold decrease after improving the protocol (table S3).

### Multiplex targeted capture

**Figure 2:**
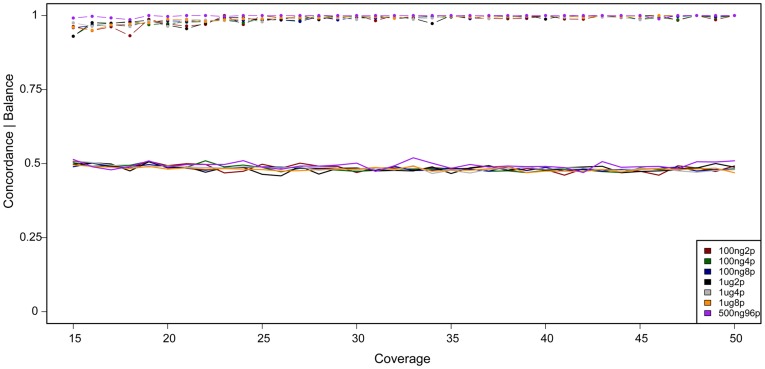
Concordance of heterozygous SNPs (lines with dots) for 100 ng and 1 µg exome libraries of different multiplexity and a 500 ng 96-plex target capture library. The average concordance for exome libraries was 99.4% with no significant difference between libraries. For the 96-plex experiment, the average concordance was 99.8%. Solid lines indicate the average allelic balance. Even in the 96-plex experiment, no bias in allelic balance is observed.

As the number of multiplexed samples increases, the concentration of the bait molecules has the potential to limit efficient capture of non-reference alleles due to competitive hybridization. To monitor such effects we prepared libraries from 1 µg of DNA and performed 2-, 4- and 8-plex captures using the SeqCap EZ Exome Library targeting 180 000 coding exons. Since sample availability is commonly limiting, we repeated the experiment using only 100 ng of DNA for library preparation. The 8-plex captures were run on a single lane on the Hiseq 2000. The 4-plex and 2-plex reactions were pooled together in 2∶1 ratios in two lanes to yield ∼1/6 lane per library. Each sample was sequenced to a mean coverage of around 42× in the target regions (figure S1). To evaluate the performance of the multiplexed capture, SNP calls were compared to variants identified using a commercially available SNP-array [29], [30]. From the sequencing data, SNPs were only called at positions with >15 in read depth that overlapped with SNPs available on the array. On average, 13328 positions were examined for each sequenced exome library. The average concordance between heterozygote (hz) variants called by the SNP-chip and the sequenced libraries was 99.4% with no significant difference between DNA input amounts or degree of multiplexing (Kruskal-Wallis, p = 0.93)(figure 2). Furthermore, we investigated the allelic bias - i.e. if the variant allele was lost in the capture step due to competitive hybridization. We could not detect any such effect (figure 2). To investigate potential biases in the modified protocol, we compared the sequences results with the standard protocol in terms of insert size, GC content and variation across targets (figure S3). We did not se any trends indicating that the modified protocol has effect on either of these parameters. For the 96-plex capture, we investigated the concordance of 94 SNPs that overlapped with our 500 kb target region and the SNP-chip. The average concordance of 2724 heterozygous SNPs across all 96 samples was 99.8% when requiring sequence coverage over 15×. As for the exome libraries, we were not able to see any evidence of a shifted allelic balance due to competitive hybridization.

## Discussion

We demonstrate that library preparation for massive parallel sequencing can be made cheap, simple and efficient. Our method is applicable on all sequencing platforms requiring addition of universal adapter handles prior to sequencing, such as Illumina, SOLiD, 454 and Ion Torrent. The absence of spin column purification makes the protocol easy to automate and reduces the loss of material. This is achieved by utilizing Taq DNA polymerase for adenylation instead of Klenow fragment exo^(−)^, which is used in the Illumina TruSeq protocol (figure 1). Klenow exo^(−)^ adds any of the four bases to 3′-ends of the DNA fragments. Therefore, nucleotides remaining from the end-repair reaction have to be removed by a clean-up step prior to adenylation. In contrast, Taq adds only dATP's even in the presence of all nucleotides, which makes a nucleotide removal step prior to adenylation superfluous. Since Taq is a thermophilic enzyme, which is inactive at low temperatures, end-polishing by T4 DNA polymerase and phosphorylation by T4 polynucleotide kinase takes place at a low temperature. Subsequently, the temperature is increased to 72°C, which allows for the adenylation reaction to start, while the mesophilic enzymes are heat-inactivated.

Targeted capture of specific genomic regions is a powerful technology for cost-efficient interrogation of limited parts of genomes. It is commonly associated with an increased manual labor to prepare the libraries required. Furthermore, in settings such as analysis of solid tumors, it is common to have a limited amount of material available for library preparation. In this study, we present a simplified laboratory procedure for preparing libraries for massively parallel sequencing. To maintain high yield while starting with a lower amount of input DNA, we changed several key aspects in the protocol. First, we changed the reaction buffer of the enzymatic steps to a single one-for-all buffer. This enabled us to remove all column-based cleanup steps in the protocol and replace them with a single cleanup step based on PEG-mediated precipitation on superparamagnetic beads. Our protocol is thus well suited for automation in any robot that is equipped with a magnet to handle superparamagnetic beads.

For studies where large numbers of samples are analyzed, the cost of preparing the libraries can be a significant proportion of the total cost. Since our protocol is based on readily available bulk enzymes, the cost is significantly reduced. To test this, we investigated the performance of three different degrees of multiplexing and evaluated the end data quality in several aspects. Firstly, the samples remain balanced after capture; i.e. a similar number of reads are sequenced from each sample in a multiplexing pool (figure S1). When increasing the number of samples in a multiplexed capture reaction, there is a risk that variant alleles are captured to a lower extent than the reference allele for which the bait was designed. However, we do not observe such effect. In our data, the allele frequency is very close to 50% in heterozygous tag-SNP positions independently of coverage (figure 2). There was no difference based on the number of samples in the multiplexing pool. Secondly, to push the number of samples in a multiplexing pool, we modified the library preparation protocol to add a specific 8-bp barcode to each well in a 96-well plate in order to perform 96-plexed capture of a genomic region of 500 kb. Even in this data, we do not see any tendency that the variant allele is captured to a lower extent (figure 2). The ability to perform multiplexing with 96 samples in parallel can cut costs for projects where large numbers of samples are analyzed significantly while maintaining individual level data.

The modifications we introduced in the protocol improved the yield of the library thus allowing us to reduce the starting amount of DNA.

## Supporting Information

Figure S1 **Average coverage in targeted regions for exome libraries. The data is even across samples even when 8 samples are pooled in the capture step.**
(TIF)

Figure S2 **qPCR plot on which the ANOVA was based.** An overnight ligation and adjusted enzyme mix significantly improve the Cy0 value in the qPCR. Each curve represents the mean of two technical replicates.
(PDF)

Figure S3 **Fold 80 base penalty (A), insert size (B) and GC-content (C) for libraries prepared with the standard and improved protocols.**
(PDF)

Table S1 **Sequences for the oligonucleotides used.**
(PDF)

Table S2 **The implications of protocol adjustments calculated using an analysis of variance table.**
(PDF)

Table S3 **Summary of sequencing data for selected libraries.** Modifying the ligation time and enzyme mix reduces the fraction of PCR duplicates approximately a factor 10-fold.
(XLSX)
