# Supplementary figures and images for: Library Preparation and Multiplex Capture for Massive Parallel Sequencing Applications Made Efficient and Easy
Source: PLoS One. 2012 Nov 5;7(11):e48616. doi: 10.1371/journal.pone.0048616 (PMC3489721; doi:10.1371/journal.pone.0048616)

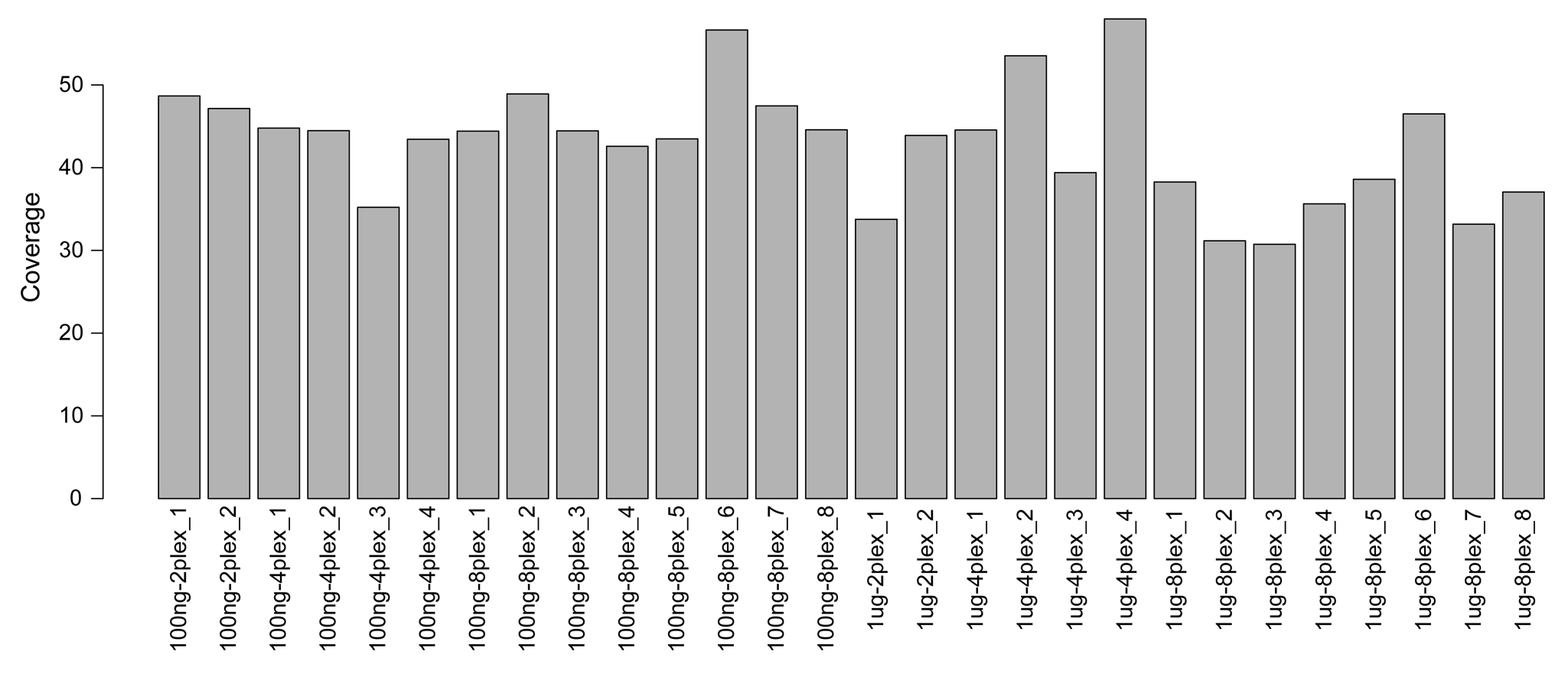

Supplement: Figure S1 — Average coverage in targeted regions for exome libraries. The data is even across samples even when 8 samples are pooled in the capture step. (TIF) [file pone.0048616.s001.tif]

# Amplification efficiency

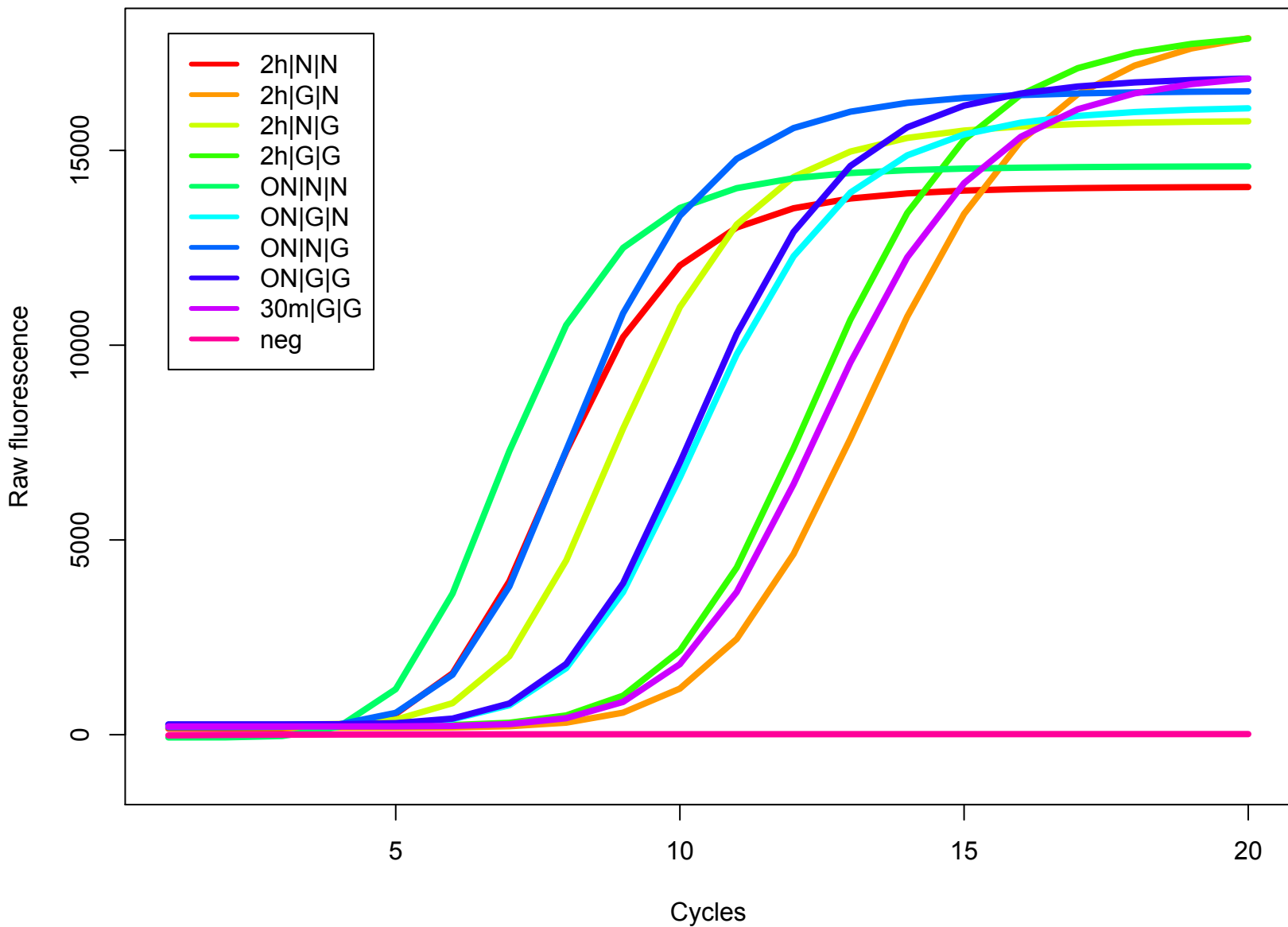

Supplement: Figure S2 — qPCR plot on which the ANOVA was based. An overnight ligation and adjusted enzyme mix significantly improve the Cy0 value in the qPCR. Each curve represents the mean of two technical replicates. (PDF) [file pone.0048616.s002.pdf]

A

## Fold 80 base penalty

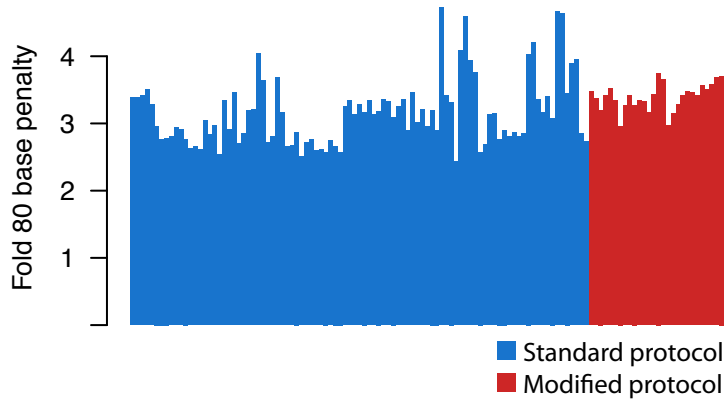

B

## Median Insert Size

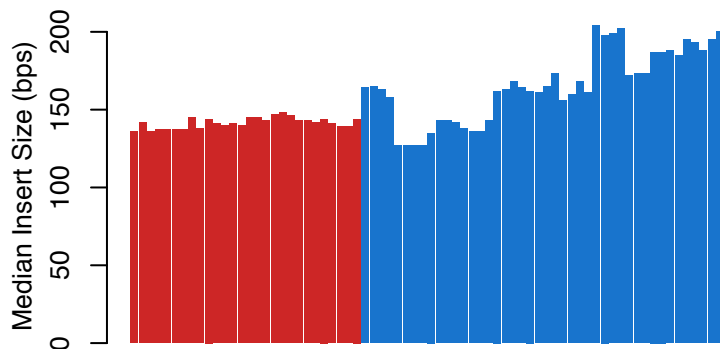

C

## Per-base GC content

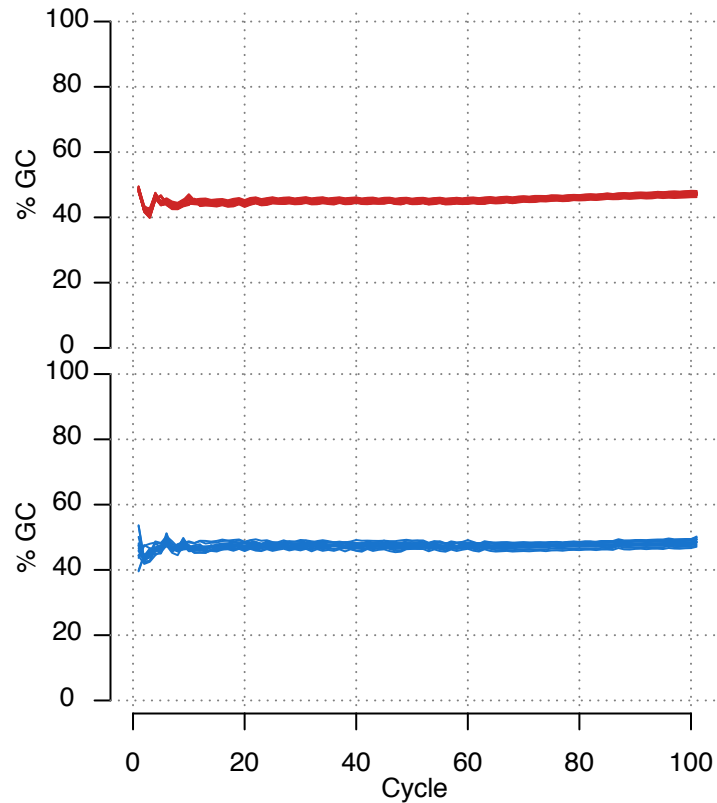

Supplement: Figure S3 — Fold 80 base penalty (A), insert size (B) and GC-content (C) for libraries prepared with the standard and improved protocols. (PDF) [file pone.0048616.s003.pdf]
