# Supplementary material for: Library Preparation and Multiplex Capture for Massive Parallel Sequencing Applications Made Efficient and Easy
Source: PLoS One. 2012 Nov 5;7(11):e48616. doi: 10.1371/journal.pone.0048616 (PMC3489721; doi:10.1371/journal.pone.0048616)
Supplement: Table S1 — Sequences for the oligonucleotides used. (PDF) [file pone.0048616.s004.pdf]

Oligo name	Sequence (5'-3')
adapter_1	ACACTCTTTCCCTACACGACGCTCTTCCGATCT
adapter_2	GATCGGAAGAGCACACGTCTGAACTCCAGTCAC
PCR_fw	AATGATACGGCGACCACCGAGATCTACACTCTTTCCCTACACGAC
PCR_indx_1	CAAGCAGAAGACGGCATACGAGATCGTGATGTGACTGGAGTTC
PCR_indx_2	CAAGCAGAAGACGGCATACGAGATACATCGGTGACTGGAGTTC
PCR_indx_3	CAAGCAGAAGACGGCATACGAGATGCCTAAGTGACTGGAGTTC
PCR_indx_4	CAAGCAGAAGACGGCATACGAGATTGGTCAGTGACTGGAGTTC
PCR_indx_5	CAAGCAGAAGACGGCATACGAGATCACTGTGTGACTGGAGTTC
PCR_indx_6	CAAGCAGAAGACGGCATACGAGATATTGGCGTGACTGGAGTTC
PCR_indx_7	CAAGCAGAAGACGGCATACGAGATGATCTGGTGACTGGAGTTC
PCR_indx_8	CAAGCAGAAGACGGCATACGAGATTCAAGTGTGACTGGAGTTC
PCR_indx_9	CAAGCAGAAGACGGCATACGAGATCTGATCGTGACTGGAGTTC
PCR_indx_10	CAAGCAGAAGACGGCATACGAGATAAGCTAGTGACTGGAGTTC
PCR_indx_11	CAAGCAGAAGACGGCATACGAGATGTAGCCGTGACTGGAGTTC
PCR_indx_12	CAAGCAGAAGACGGCATACGAGATTACAAGGTGACTGGAGTTC
