# Supplementary material for: Library Preparation and Multiplex Capture for Massive Parallel Sequencing Applications Made Efficient and Easy
Source: PLoS One. 2012 Nov 5;7(11):e48616. doi: 10.1371/journal.pone.0048616 (PMC3489721; doi:10.1371/journal.pone.0048616)
Supplement: Table S2 — The implications of protocol adjustments calculated using an analysis of variance table. (PDF) [file pone.0048616.s005.pdf]

Source	Partial SS	df	MS	F	Prob > F
Model	60.48	3	20.16	47.3	0.0000
over-night ligation	11.11	1	11.11	26.06	0.0003
adjusted enzyme mix	48.97	1	48.97	114.88	0.0000
adjusted incubation	0.41	1	0.41	0.97	0.3445
Residual	5.11	12	0.43		
Total	65.60	15	4.37		
